# Metabolic Pathways in Alloreactive T Cells

**DOI:** 10.3389/fimmu.2020.01517

**Published:** 2020-07-24

**Authors:** Rebecca A. Brown, Craig A. Byersdorfer

**Affiliations:** Division of Blood and Marrow Transplant and Cellular Therapies, Department of Pediatrics, University of Pittsburgh School of Medicine, Pittsburgh, PA, United States

**Keywords:** alloreactive T cells, GVHD biology, immunometabolism, glycolysis, fatty acid oxidation (FAO), mammalian target of rapamycin (mTOR), AMPK

## Abstract

Allogeneic hematopoietic stem cell transplantation (aHSCT) is a curative therapy for a range of hematologic illnesses including aplastic anemia, sickle cell disease, immunodeficiency, and high-risk leukemia, but the efficacy of aHSCT is often undermined by graft-versus-host disease (GVHD), where T cells from the donor attack and destroy recipient tissues. Given the strong interconnection between T cell metabolism and cellular function, determining the metabolic pathways utilized by alloreactive T cells is fundamental to deepening our understanding of GVHD biology, including its initiation, propagation, and potential mitigation. This review summarizes the metabolic pathways available to alloreactive T cells and highlights key metabolic proteins and pathways linking T cell metabolism to effector function. Our current knowledge of alloreactive T cell metabolism is then explored, showing support for glycolysis, fat oxidation, and glutamine metabolism but also offering a potential explanation for how these presumably contradictory metabolic findings might be reconciled. Examples of additional ways in which metabolism impacts aHSCT are addressed, including the influence of butyrate metabolism on GVHD resolution. Finally, the caveats and challenges of assigning causality using our current metabolic toolbox is discussed, as well as likely future directions in immunometabolism, both to highlight the strengths of the current evidence as well as recognize some of its limitations.

## Introduction

Allogeneic hematopoietic stem cell transplantation (aHSCT) is a potentially curative therapy for a wide range of hematologic maladies ranging from genetic diseases to aggressive leukemias and lymphomas (1–3). An unintended and potentially deadly consequence of aHSCT is graft-versus-host disease (GVHD), where donor T cells primed to react against alloantigens attack host tissues in the skin, gastrointestinal tract, and liver (4). While corticosteroid-induced immunosuppression can treat GVHD, corticosteroids are an imperfect therapy and durable remissions only occur in 50% of patients (5). Furthermore, the broad immunosuppression necessary to treat GVHD often limits physiologic immunity and impairs T cell mediated clearance of leukemia [aka the graft-versus-leukemia (GVL) effect (6, 7)]. Thus, common complications following the increased immunosuppression surrounding GVHD treatment include infection (8, 9) and cancer relapse (9), making development of GVHD especially dangerous. In fact, while absolute T cell depletion can minimize GVHD risk, the ensuing increase in infection, and disease relapse result in comparable overall survival (10). Thus, there is a need for novel approaches to treat and prevent GVHD while still preserving physiologic immunity and maintaining aHSCT efficacy.

It is broadly accepted that metabolism and immune cell function are linked, with immunologic differentiation influencing immune cell metabolism and metabolic pathways impacting immune responses (11, 12). It is therefore imperative to understand how metabolism influences T cell function in specific environmental contexts. This is particularly true during GVHD, where high levels of chronic antigen stimulation result in robustly activated T cells with a sharply increased metabolic demand. Understanding the unique metabolic profile of alloreactive T cells will enhance our ability to improve current therapeutic options and advance our contextual knowledge of *in vivo* T cell biology. This review will highlight our current understanding of alloreactive T cell metabolism in light of the major metabolic pathways, present evidence for involvement of various pathways at distinct stages of the process, define key metabolic regulators that influence substrate choice, and integrate multiple lines of evidence into a cohesive overarching hypothesis. We close by highlighting examples of additional ways in which metabolism can influence GVHD and discuss challenges to the interpretation of metabolic data.

## Overview of Cellular Metabolism

Cellular metabolism is a complex interplay between multiple different enzymes, substrates, intermediates, and end products. Classically, glycolysis, and oxidative phosphorylation (OXPHOS) have been studied as the primary pathways that supply cellular energy. Glycolysis consists of a series of enzymatic steps that convert glucose into pyruvate. Depending on the intrinsic and extrinsic needs of the cell, pyruvate can then either be converted into lactate and excreted from the cell or channeled into acetyl-coA and further oxidized via OXPHOS. While lactate fermentation classically occurs in oxygen poor environments, T cells can perform glycolysis and lactate fermentation in oxygen replete environments, referred to as aerobic glycolysis. Although glycolysis might not be the best choice based solely on energy production, glycolytic intermediates can also act as substrates for anabolic pathways including amino acid synthesis, nucleotide synthesis, and the pentose phosphate pathway (PPP) (13), all processes necessary in actively proliferating cells.

Oxidative phosphorylation is a more efficient process used to generate cellular energy. Specifically, the tricarboxylic acid (TCA) cycle uses the end products of glycolysis, fatty acid oxidation, and glutamine metabolism to generate the reducing intermediates, NADH, and FADH_2_ (14). NADH and FADH_2_, in turn, fuel the electron transport chain (ETC) by donating electrons to Complex I and II (14), a process which results in ATP production and concurrent consumption of oxygen (14).

## Metabolic Pathways Contributing to Alloreactive T Cell Effector Function

Classically, naïve T cells are considered largely quiescent, catabolically relying on OXPHOS to meet their modest energy demands. Upon activation, naïve T cells switch to anabolic metabolism (15) and despite the availability of oxygen, increase aerobic glycolysis in a process known as the Warburg effect (16, 17). Aerobic glycolysis produces less energy per molecule of substrate than oxidative pathways but has the advantage of maintaining redox balance (18) and allowing for the bulk of cellular machinery to be used in the production of biomolecules required for proliferation and T cell function, including cytokines (19, 20). In contrast to recently activated T cells, memory and regulatory T cells (T_regs_) rely on oxidation of fatty acids and glucose to maintain their energetic balance (21–25). This classic view has recently been challenged, where effector T cells have been demonstrated to increase oxidative metabolism *in vivo* and be less reliant on glycolytic metabolism compared to *in vitro* activated cells (26). How alloreactive T cells meet their energetic demands during GVHD remains a work in progress, but evidence supports the adoption of both aerobic glycolysis and OXPHOS during early stages of T cell activation and disease initiation.

The studies highlighted in this review compare the profile of allogeneic T cells to either syngeneic or naïve T cell controls. While both syngeneic and naïve T cells are less activated than alloreactive cells, syngeneic T cells are the preferred negative control because they experience the inflammatory milieu of pre-transplant conditioning and most accurately reflect the lymphopenia-driven reconstitution of the immune system seen in human transplants. In contrast, naïve T cells are relatively inert. Thus, comparing alloreactive and naïve T cells risks identifying differences that are not unique to alloreactive T cells, but are instead characteristic of any proliferating T cell. Since the aim is to distinguish alloreactive from homeostatically proliferating T cells, syngeneic T cells remains the better negative control.

Since aerobic glycolysis is critical for physiologic T cell activation (16, 27, 28), it is natural to consider the role of glucose in alloreactive cells. However, discussions on glucose metabolism are challenging given that pyruvate, a critical glycolytic intermediate, can either be converted into lactate for fermentation or alternatively channeled through pyruvate dehydrogenase into the TCA cycle for oxidation. For the purposes of this review, we will consider both forms of glucose metabolism under one discussion. In two pre-clinical models of GVHD, glucose uptake increased in donor T cells 14 days post-transplant (allogeneic > syngeneic), accompanied by increased expression of glucose transporters Glut1 and Glut3 (29). Expression of the key glycolytic enzymes hexokinase (isoforms 1 and 2) and lactate dehydrogenase also increased in allogeneic cells, while donor T cell numbers decreased following treatment with the pan-glycolysis inhibitor 2-deoxyglucose (2DG) (29). In this same study, recipients experienced improved survival when treated with 3-(3-pyridinyl)-1-(4-pyridinyl)-2-propen-1-one (3-PO), an inhibitor of phosphofructokinase (30), the rate-limiting step in glycolytic metabolism (29, 31, 32). Consistent with glucose transport playing an important role in GVHD development, T cells lacking *Glut1*, which experienced a decreased glycolytic rate *in vitro*, were unable to induce GVHD *in vivo*. In this case, post-transplant weight loss and survival outcomes were similar between recipients of Glut1^−/−^ T cells and those transplanted with bone marrow alone (33). In addition to its therapeutic potential, increased glucose uptake may also have diagnostic implications. Positron emission tomography using ^18^F-fluorodeoxyglucose (FDG-PET), a glucose analog, identifies increased tracer uptake in the gastrointestinal tract during GVHD in both mice and humans (34). Thus, inhibiting glycolytic metabolism, either genetically or pharmacologically, might constrain alloreactive T cell effector (T_eff_) function, while increased glucose trafficking may be of potential diagnostic value. The caveat is that glycolytic metabolism may also prove essential to the function of non-GVHD T cells.

A key metabolic regulator that could connect glycolytic metabolism and alloreactive T cell function is the mammalian target of rapamycin (mTOR), a serine/threonine kinase belonging to the phosphoinositol 3-kinase (PI3K) family. mTOR is the catalytic subunit for either of two distinct protein complexes; mTORC1 (with scaffolding protein Raptor) and mTORC2 (with scaffolding protein RICTOR) (35). mTORC1 and mTORC2 each have unique roles within cells. mTORC1 promotes protein and lipid synthesis while mTORC2 promotes cytoskeletal rearrangement (36). In a variety of settings, mTOR integrates environmental signals into regulation of immune cell metabolism, differentiation, and effector function. In particular, mTOR and has been shown to play a key role in T cell activation and cell fate (37–39). Metabolically, T cell receptor stimulation activates mTOR via PI3K/Akt signaling, which then promotes glycolysis, glutaminolysis, and activation of the PPP (37, 40, 41). mTOR promotes glycolysis in part through the activation of HIF1α and c-Myc, transcription factors which drive expression of glycolytic proteins including pyruvate dehydrogenase kinase 1, hexokinase 2, and lactate dehydrogenase A (40, 42–44). Furthermore, the PI3K/Akt/mTORC1 pathway has been implicated in T cells as a key regulatory step for the expression and trafficking of the glucose transporter, Glut1, with mTORC inhibitors preventing Glut1 expression (33, 45). Thus, mTOR contributes to metabolic reprogramming following T cell activation by promoting both glycolysis and general anabolic pathways. mTOR signaling also influences T cell differentiation. T cells deficient in mTOR are unable to differentiate into T-helper type 1 (Th1), Th2, or Th17 cells under *in vitro* skewing conditions but still readily differentiate into FoxP3^+^ T_reg_ cells (46). Furthermore, mTORC1, and mTORC2 promote differentiation of specific T cell subsets. mTORC2 promotes Th2 differentiation (47), while mTORC1 promotes development of Th17 cells (47, 48). The role played by mTORC1 in Th1 differentiation remains uncertain, with evidence both for (47), and against (48) mTORC1 involvement in Th1 responses.

mTOR has also been implicated in promoting the pathogenicity of alloreactive T cells. In pre-clinical models, mTOR activity increases in alloreactive T cells and both the pharmacologic inhibition of mTOR, and its genetic deletion, inhibit glycolysis without impacting OXPHOS (29, 49). These interventions also improve outcomes in animal models of GVHD (29). In the clinic, targeting mTOR using the inhibitors sirolimus (rapamycin) and everolimus is well-established and has been found to be efficacious for both GVHD prophylaxis and treatment (50, 51). Thus, inhibition of mTOR improves GVHD, in part through its inhibition of glycolysis.

In addition to glucose metabolism, there is ample evidence that alloreactive T cells rely on the energy and by-products of OXPHOS. This necessity of OXPHOS, in addition to an increase in lactate fermentation, is likely necessary due to the greater energetic demands experienced by T cells undergoing constant exposure to high levels of antigen in a near continuous manner. In allogeneic cells, oxygen consumption increased markedly compared to either naïve T cells or T cells recovered from syngeneic recipients (52). Mitochondrial activity also increased in allogeneic T cells with a corresponding increase in mitochondrial superoxide production (52, 53). Consistent with increased mitochondrial activity, alloreactive T cells upregulated expression of peroxisome proliferator-activated receptor gamma coactivator 1-alpha (Pgc1α), a regulator of mitochondrial biogenesis (54). Finally, administration of BZ-423, an F1-F0 ATPase inhibitor that targets cells with increased mitochondrial respiration, improved survival, lowered clinical scores, and decreased lymphocytic infiltration into GVHD target organs in a murine model of GVHD (53). These results suggest that alloreactive T cells are preferentially susceptible to ETC inhibition, in part because of their increased reliance on mitochondrial respiration (53). Taken together these findings demonstrate an increased dependence on both OXPHOS and glycolytic metabolism in alloreactive T cells, making them metabolically distinct from other T cell populations.

Although the mechanisms that influence OXPHOS are complex, the cellular energy sensor AMP-activated protein kinase (AMPK) has been implicated as a driver of oxidative metabolism and could play a role. AMPK is heterotrimeric protein complex consisting of a serine/threonine kinase α subunit, a stabilizing β subunit, and a regulatory γ subunit. The γ subunit detects low intracellular energy levels by sensing the AMP/ATP ratio (increased during low energy states) and responds by facilitating activation of the AMPKα kinase domain (55). AMPK activation conserves energy by inhibiting anabolic pathways (e.g., fat and protein synthesis), while increasing energy production via catabolic pathways, including OXPHOS and autophagy (56, 57). In part, AMPK restricts anabolism by antagonizing mTORC1 via phosphorylation of Raptor as well as upstream regulator tuberous sclerosis complex 2 (28, 58). AMPK also directly promotes OXPHOS and fatty acid catabolism. In skeletal muscle, acetyl-coA carboxylase (ACC) produces malonyl-coA, which allosterically inhibits carnitine palmitoyl transferase 1 (CPT1a), a key enzyme in fat oxidation, and thereby blocks FAO (55, 59). AMPK inhibits ACC through phosphorylation, which decreases malonyl-CoA levels and thus increases FAO. In addition, ACC is a key enzyme in fatty acid synthesis (FAS) so that AMPK inhibition of ACC not only increases FAO but also reduces the anabolic process of FAS.

Given that AMPK is key to promoting both OXPHOS and FAO, it follows that AMPK would be integral to T cell homeostasis. However, the exact role for AMPK in T cells continues to evolve. Systemic ablation of AMPKα1 increased lymphocyte susceptibility to mitochondrial inhibition (i.e., treatment with oligomycin) but did not impact T cell development or differentiation (60). Later studies noted an increase in T cell glycolysis following global deletion of AMPKα1 and an increase in T cell production of the pro-inflammatory cytokines interferon gamma (IFN-γ) and interleukin 17a (IL-17a) (61). In a *Listeria monocytogenes* infection model, T cell-specific deletion of AMPKα1 impaired memory CD8 cell generation compared to wildtype T cells without impacting primary immune responses (62). More recently, AMPK was shown to be necessary for maximal T_eff_ generation during both viral and bacterial challenges *in vivo* (63) and has been implicated in driving oxidative metabolism in T cell acute lymphoblastic leukemia (64). AMPK has also been suggested to be necessary for T_reg_ development, with increased AMPK phosphorylation in cultured T_reg_ cells and an increase in T_reg_ percentages following *in vivo* administration of metformin, an indirect AMPK activator (24). Metformin also increases T_reg_ number at the expense of Th17 cells in models of autoimmune arthritis (65, 66). However, whether AMPK is directly responsible for these changes, or they are driven by actions of metformin independent of AMPK, remains to be determined.

Despite characterization of AMPK in activated T cells, how AMPK contributes to GVHD pathogenicity is poorly understood. In the only paper to date investigating AMPK and GVHD, 5 days of metformin administration ameliorated GVHD severity and decreased disease lethality, with fewer Th17 and Th1 and increased T_reg_ cells recovered from metformin-treated recipients (49). However, given the fact that metformin is a direct inhibitor of Complex I of the ETC (67–69), there is a high likelihood that metformin directly inhibits oxidative metabolism, a process necessary in alloreactive cells. In our hands, transplantation of donor T cells lacking both AMPK α1 and α2 improved GVHD-related lethality with decreased recovery of AMPK-deficient donor T cells, reduced T cell homing to target organs, and an improved T_reg_ to T_eff_ cell ratio (70). Indeed, these later results are in line with studies demonstrating an important role for AMPK in T_eff_ cell survival and recovery (63). Thus, on balance, metformin treatment improves GVHD, but likely in an AMPK independent manner, as genetic elimination demonstrates that AMPK is necessary in donor T cells for maximal GVHD severity. In both cases, more work needs to be done to determine the exact mechanism behind the observed effects.

Given the increased oxidative metabolism of donor T cells during GVHD, an ongoing question becomes which substrate or substrates fuel this pathway. In fact, glucose utilization, fat oxidation, and glutamine metabolism have all been shown to be crucial for T cell proliferation and survival in various allogeneic contexts (15, 71, 72). T cells isolated on day 7 post-allogeneic transplant show elevated levels of fat import, higher acylcarnitine concentrations, and increased fatty acid oxidation (53, 54). These changes are supported by increased expression of CPT1a and CPT2, enzymes necessary for transport of long and very-long chain fatty acids into the mitochondria for β-oxidation. Furthermore, treatments with the FAO inhibitor etomoxir improved GVHD severity while simultaneously decreasing T cell proliferation and the number of donor T cells (54). This dependence on FAO may be most prevalent at early times post-transplant, as metabolic interrogation at later time points, in a distinct model of GVHD, demonstrated fat transport in allogeneic cells at an intermediate level between unstimulated and syngeneic T cells (29). Thus, the timing of FAO in alloreactive T cells, as well as its absolute necessity, remains unresolved.

In addition to lipids and glucose, glutamine is another common metabolic substrate for T cells. From the beginning, glutamine uptake and metabolism have been shown to increase following T cell activation and glutamine is required for both Th1 and Th17 differentiation (71, 73). Glick et al. demonstrated that glutamine can act as an anaplerotic nutrient source in alloreactive T cells to replenish TCA cycle intermediates and provide substrates for the PPP (74). However, in contrast to the glutamine dependence seen in alloreactive cells, which would suggest worsening disease with glutamine supplementation, there is strong evidence that systemic glutamine administration facilitates therapeutic recovery following aHSCT. In a murine model of GVHD, systemic glutamine administration increased T_reg_ numbers and decreased serum levels of tumor necrosis factor α, limiting pro-inflammatory immune responses and improving recipient survival by 30% (75). In human patients, glutamine supplementation improved post-transplant survival with a trend toward decreased rates of GVHD (76). Thus, while there is much to be learned regarding glutamine metabolism in individual cell types, systemic glutamine administration appears to be protective.

## Metabolism in Regulatory T Cells

T_regs_ provide crucial inhibitory signals to T_eff_ and are key to dampening immune responses and promoting tolerance (77). In GVHD, T_regs_ are of immense interest because of their potential ability to correct the balance between inflammation and immunosuppression. In fact, the frequency of T_reg_, as marked by CD4^+^CD25^+^ expression, was lower in patients with chronic GVHD than in healthy controls or in patients post-transplant without chronic GVHD (78). Furthermore, enhancing T_reg_ frequency, either through ultra-low dose IL-2 administration, or adoptive transfer of *ex vivo* expanded T_reg_, is an effective way to improve GVHD (79–81). Thus, increasing T_reg_ frequency could be an essential component for GVHD prevention and treatment and metabolic interventions could play a large role in this effort.

In addition to filling a unique immunologic niche, T_regs_ have a metabolic profile distinct from T_eff_ cells. T_regs_ generated *in vitro* increase their reliance on lipid and mitochondrial metabolism (24) while transgenic expression of the Glut1 receptor increases glycolysis, which impedes suppressive function. Opposing this signaling is the transcription factor Foxp3, which reprograms T_reg_ metabolism toward OXPHOS and away from glycolysis (82, 83), driving up T_reg_ suppressive capacity. Foxp3 is thought to achieve these results by inhibiting Myc (82), a transcription factor that promotes both glycolysis and glutamine metabolism (43). Thus, changes in suppressive function are metabolically dependent, with a loss of OXPHOS decreasing suppressor activity (84). This tuning of T_reg_ suppressive capacity may be integral to the propagation and subsequent waning of an immune response. At times of high stimulation (e.g., infection) T_reg_ increase in number but are minimally suppressive, allowing effector responses to proceed unabated. As the infection subsides, inflammatory signals decrease and FoxP3 levels stabilize, leading to the adoption of OXPHOS, which decreases T_reg_ proliferation but increases suppressive function, limiting the effector response and restoring a state of tolerance (83).

Multiple studies have also demonstrated an integral link between mitochondrial metabolism and T_reg_ mediated suppression both *in vitro* and *in vivo* (84–86). Deletion of complex III specifically in T_reg_ led to development of a fatal early inflammatory disease (87) and transfer of Complex III deficient T_reg_ was unable to protect recipients in a model of T-cell driven colitis (85). T_reg_ also depend upon the mitochondrial transcription factor A (Tfam), which controls mitochondrial DNA copy number and is integral to ETC activity (88, 89). Loss of Tfam in T_reg_ decreased mitochondrial respiration, blunted expression of inhibitory markers ICOS and CTLA4, and resulted in a severe inflammatory disorder (86). Interestingly, although mTOR signaling is known to drive T cell glycolysis (which is expected to decrease T_reg_ function), mTOR is also required for proper T_reg_ function and development. Compared to conventional T cells, T_reg_ have higher mTORC1 activity (90, 91) and T_reg_-specific deletion of Raptor, an obligate component of the mTORC1 complex, resulted in a fatal inflammation and loss of T_reg_ suppressor function (91). Similar results were found in mice lacking the mTOR protein, where Th2 responses increased significantly in the lungs and gastrointestinal tract of knock-out mice (86).

Despite our increased working knowledge of T_reg_ metabolism, little is known about T_reg_ metabolism in the alloreactive environment. Sirtuin-1 (Sirt1) is a class III histone deacetylase whose expression influences multiple metabolic pathways. Donor T cells lacking Sirt1 show increased FoxP3 stability in inducible T_reg_ (iT_reg_) with a subsequent decreased conversion to pathogenic IFN-γ producing cells and a loss of follicular helper T cell development (92). In other studies, human iTregs propagated *in vitro* via pSTAT3 inhibition prevented xenogeneic GVHD yet spared donor antileukemia immunity. Metabolically, pSTAT3 inhibition shifted iTreg metabolism from OXPHOS to glycolysis, with a reduction in ETC activity. However, this metabolic impairment could be corrected by treating pSTAT3-inhibited T_reg_ with coenzyme Q10, which restored OXPHOS and augmented their suppressive potency (93). In other work, adoptive transfer of T_reg_ lacking vimentin, or pre-treated with the phosphokinase C inhibitor AEB071, improved GVHD survival, clinical scores, and weight loss to a greater degree than WT T_reg_. Mechanistically, absence or inhibition of vimentin enhanced oxidative metabolism within the T_reg_ compartment and concomitantly increased their suppressive capacity (94). Finally, transplantation of adenosine producing CD150^+^ T_reg_ into allogeneic animals decreased the severity of immune cell infiltration into the intestine (95). Thus, T_reg_ function is a finely tuned process, accomplished through integration of multiple inputs including mTOR signaling, intracellular energy sensing, metabolic pathways, and the influence of local environmental cues including danger signals. Furthermore, T_reg_-associated metabolic changes found in other contexts appear to hold following aHSCT, in particular the association between increased OXPHOS and enhanced suppressive capacity.

## Effects of Metabolic Inhibition on Graft-vs.-Tumor Responses

A major indication for allogeneic transplantation is relapsed or refractory leukemia and lymphoma. aHSCT's benefit in this setting derives from donor T cells reactivity against foreign tumor cells, the so-called Graft-versus-tumor (GVT) effect. It is expected that anything that interrupts T cell alloreactivity, or impairs allogeneic T cell number, might disrupt the therapeutic efficacy of allogeneic transplantation. And yet metabolic manipulation does not appear to be universally detrimental to anti-cancer responses. Donor T cells that lack AMPK induce less severe GVHD but continue to demonstrate preserved or even enhanced cytotoxic potential post-transplant (70). Similarly, mice treated with recombinant Thioredoxin at the time of transplantation exhibit decreased GVHD severity while simultaneously preserving GVT effects (96). In some cases, preserved cytotoxicity results from a preservation in cytokine responses in remaining T cells (70), coupled with a decrease in T cell dysfunction due to lower rates of GVHD (97). In other cases, better tumor control may result from the dual impact of metabolic modulation on both alloreactive T cells and the underlying malignancy (98, 99). It has also been argued that T cell activation, and hence metabolic demands, operate on a continuum, with GVHD-causing T cells at the far end of the activation and metabolic spectrum (100). In this case, highly active T cells would be more susceptible to metabolic or similar perturbations than anti-tumor T cells with more modest energy requirements. Indeed, Treg transfer experiments support this concept of differential sensitivity, as exogenous Treg administration sufficiently controls alloreactive T cell expansion without compromising GVT activity (101).

## Metabolic Influence Beyond T Cells

It was been known for some time that T_reg_ induction in the gastrointestinal tract is influenced by production of short chain fatty acids (SCFAs), commonly produced by commensal bacteria, and primarily in the forms of butyrate and propionate. SCFAs induce Foxp3 expression (102, 103) by either inhibiting histone deacetylases or by activating G-protein receptor 43 (GPR43) (103). Butyrate levels were found to be excessively low in intestinal tissues following allogeneic transplantation and administration of exogenous butyrate increased these levels back to normal while decreasing GVHD severity and improving weight loss and clinical scores (104). However, these beneficial effects were found to be independent of T_reg_ and instead resulted from direct salutary effects of butyrate on the intestinal epithelial cells (IECs), essentially improving the host response to injury. In a follow-up study, GPR43 expression was found to be necessary to realize the GVHD-protective effects of butyrate and because of this necessity GVHD severity increased in mice lacking GPR43 (105). Ultimately, GPR43 signaling increased inflammasome activation in IECs and enhanced IEC integrity and epithelial repair secondary to increases in local cytokine secretion including IL-18. Thus, metabolites and metabolic pathways beyond those utilized directly by T cells can have a profound effect on GVHD pathobiology, in part by influencing host cell responses.

## Toward a Unifying Theory of Alloreactive T Cell Metabolism

**Figure 1:**
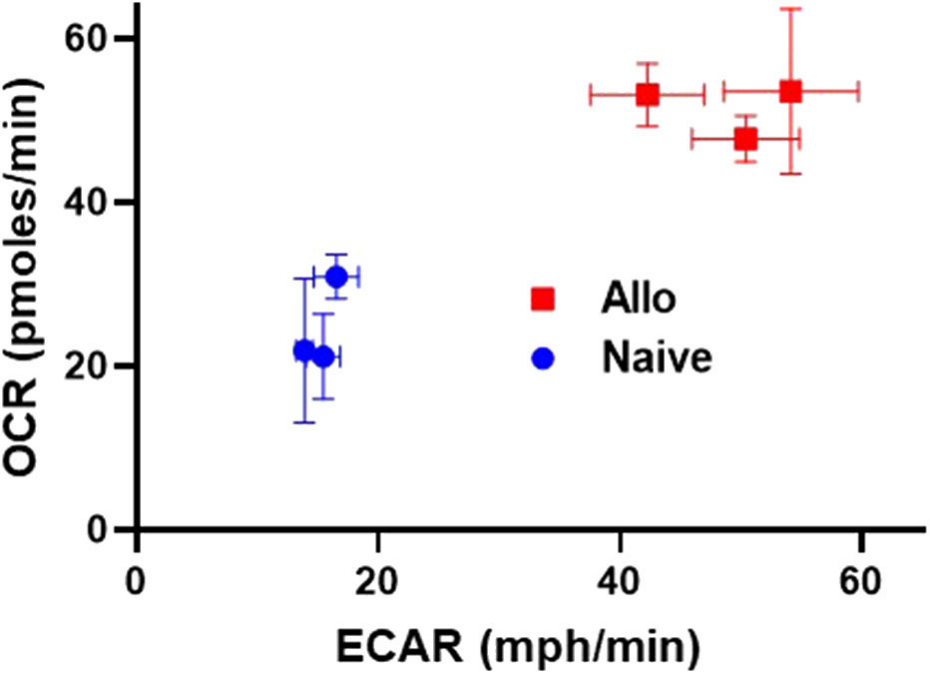
Alloreactive CD8^+^ T cells simultaneously increase both OXPHOS and glycolysis. 2 × 10^6^ CD45.1^+^ B6 T cells and 5 × 10^6^ B6 bone marrow (BM) cells were transplanted into irradiated, allogeneic (B6 × DBA F1) recipients. On day 7 post-transplant, donor CD8^+^ T cells (CD8^+^CD45.1^+^TCR-β^+^) were flow-sorted and 2 × 10^5^ cells placed into a Seahorse metabolic analyzer. Values for both oxygen consumption rate (OCR) and extracellular acidification of the media (ECAR), a proxy for glycolysis, were simultaneously increased in day 7 CD8^+^ donor T cells.

As highlighted thus far, alloreactive T cell metabolism is complex, with studies implicating a role for multiple and sometimes opposing metabolic pathways and substrates. While some contradictory findings might relate to minor differences in animal models, or the time point tested, an additional possibility is that the metabolic pathways being considered are not mutually exclusive and alloreactive T cells might upregulate multiple pathways at the same time. To this point, data from our lab demonstrates that CD8 T cells isolated from allogeneic recipients on day 7 post-transplant simultaneously increased both OXPHOS, as measured by oxygen consumption rates, and glycolysis, as measured by extracellular acidification (Figure 1). These data argue that OXPHOS and glycolysis are not mutually exclusive pathways within T cells and instead hint that alloreactive T cells might increase both aerobic glycolysis and OXPHOS simultaneously to meet their increased energy needs.

## Current Challenges

Inherently, our understanding of T cell metabolism is limited by the availability of current methods—namely pharmacologic manipulation and genetic deletion. While both tools can determine the role of a particular target in a specific pathway or disease process, pharmacologic manipulation and genetic deletion both come with limitations. For pharmacologic activators and inhibitors, this limitation is often an unintended effect of the drug, whereas genetic knockouts are often compromised by timing of the deletion in relation to when the effects are being measured.

Looking more closely at pharmacologic manipulation, activators and inhibitors can work indirectly, have off-target effects, or mediate on-target effects in off-target cells/tissues. The use of metformin as an AMPK activator is a perfect example of this challenge (106–108). Because metformin inhibits complex I of the ETC, it activates AMPK *indirectly* by increasing the AMP:ATP ratio (109–111). Thus, it is often difficult to determine if metformin-related changes are due to AMPK activation or instead to metformin's direct effects on the ETC. Inhibitors can also cause off target effects. Many foundational studies used the CPT1a inhibitor etomoxir to study FAO (112, 113) and connect FAO with specific changes in T_reg_ and/or memory T cell (T_mem_) populations (24, 25, 114). Recently, however, it was shown that the higher concentrations of etomoxir used in most studies not only inhibited long-chain FAO, but also broadly inhibited OXPHOS secondary to a decreased abundance of TCA cycle intermediates (115). Thus, given the potential off-target effects of etomoxir, interpretation of many seminal findings have come under increased scrutiny. Lastly, pharmacologic administration can cause unwanted effects in off-target tissues, resulting in toxic side effects and limiting the utility of the drug. For example, the glutamine antagonist, 6-Diazo-5-oxo-L-norleucine (DON) (116), promotes tolerance in combination with glycolytic inhibitors (117). However, its use has been limited because of severe on target, off-tissue toxicity, particularly to the gastrointestinal tract (118, 119). This challenge has led to the development of a DON pro-drug to more specifically deliver glutamine inhibition directly to tissues of interest (120).

Regarding genetic knockouts, while enhanced technologies can target a specific gene of interest within a given tissue type, timing of the deletion, particularly vis-à-vis measurement of effect, becomes extraordinarily important. In most cases of T cell specific-deletion, Cre recombinase is expressed under control of either the CD4 or lymphocyte protein kinase (Lck) promoter. In both cases, genetic deletion occurs during T cell development, leaving a long time for T cells to utilize alternative compensatory pathways, akin to taking the back-roads into work when the highway is unavailable. This point is important to keep in mind, as experiments using genetic knockouts might easily yield results confounded by the adoption of compensatory pathways.

In the example cited earlier, Raud et al. (115) used CD4 promoter driven Cre expression to delete CPT1a specifically in T cells, allowing them to conclude that T_mem_ and T_reg_ cells developed *in vitro* and *in vivo* in the absence of CPT1a (presumably lacking FAO). While these findings appear to contradict a necessity for FAO in T_reg_ and T_mem_ cells (24, 25, 114), alternative explanations exist. A distinct possibility is that an extended loss of CPT1a allowed T cells to become dependent on other metabolic pathways (121). In fact, Raud et al. (115) concede that their experiments did not limit medium or short-chain FAO, leaving open the possibility of this alternative pathway. In this one example, acute inhibition of FAO (as would be the intent with pharmacologic inhibitors) might give a very different outcome than prolonged absence of fat oxidation using genetic models, highlighting that timing of genetic deletion vis-à-vis measurement of effect must be strongly considered in every situation.

The potential for metabolic flexibility also influences our approach in treating T cell-driven pathogenesis. For example, treatment efficacy could improve by targeting multiple metabolic pathways simultaneously, in essence restricting T cells from upregulating compensatory pathways. Akin to retroviral therapy for human immunodeficiency virus (HIV) (122, 123), where a combination of drugs targets different viral components, targeting multiple metabolic pathways concurrently may be necessary to overcome the metabolic adaptations of pathogenic cells. In the context of solid organ transplantation, the combination DON (glutamine inhibition), 2-DG (glycolytic inhibition), and metformin (targeting OXPHOS) effectively promoted tolerance in fully mismatched skin and heart allograft models (117). Alternatively, inhibiting a mediator central to the metabolic reprogramming of multiple pathways might also be feasible. During GVHD, PD-L1 was shown to be central to reprogramming multiple T cell pathways and PD-L1 deficient T cells reduced glycolysis, OXPHOS, and FAO, improving GVHD outcomes in the process (124).

Finally, careful study of T cell metabolism as well as secondary/tertiary compensatory pathways will improve the timing and specificity of our inhibition. In this regard, aHSCT offers the distinct advantage of having a period in which donor cells are manipulated *ex vivo* prior to transplantation into recipients. Leveraging this advantage, metabolic inhibitors or activators could be applied exclusively to donor cells, sparing tissues from systemic administration of the compound. Furthermore, it may be possible to inhibit a first pathway through *ex vivo* manipulation (e.g., FAO), followed by subsequent inhibition of a compensatory pathway *in vivo* through systemic administration of a second inhibitor (e.g., glutamine metabolism). Cells lesioned in the first pathway would be expected to be more sensitive to secondary inhibition, while T cells arising *de novo* (and not having experienced the primary inhibition) would be spared. Ultimately, precise metabolic modulation and the potential for simultaneous inhibition of multiple metabolic pathways, will enhance the efficacy of metabolism-based therapies.

## Future Directions

The breadth of research highlighted thus far lays a strong foundation for an increased understanding of T cell metabolism during aHSCT. However, outstanding questions remain regarding the heterogeneity of T cells recovered post-transplant and whether murine findings are translatable to humans. Like many complex disease processes, T cells collected and analyzed post-aHSCT represent a heterogenous population of cells including those driving the alloreactive response and bystanders simply responding to the inflammatory milieu. Studying metabolism in whole T cells during GVHD thus glosses over differences between individual T cell subsets and complicates the understanding of metabolism in the most highly alloreactive T cells. One approach to clarify this issue has been to use GVHD models in which donor T cells respond to a specific antigen. For example, transgenic CD8 T cells that recognize the SIINFEKL peptide of ovalbumin (i.e., OT-1 T cells) can be injected into CAG-OVA recipients which express ovalbumin as a self-antigen. Using this model, T cells transplanted into CAG-OVA recipients expressed increased levels of ROS and PD-1 compared to OT-1 T cells responding to immunization with CAG-OVA expressing dendritic cells (125). In a different study, OT-1 T cells isolated during GVHD increased fat transport while bystander (non-OVA reactive) T cells did not (54). However, the use of transgenic systems for the study of metabolism in alloreactive T cells has been limited.

Another approach is to use single-cell metabolomics to determine the individual metabolic profiles of donor T cells isolated from allogeneic recipients, a breakthrough that has the potential to revolutionize the study of immunometabolism during GVHD. While there are many groups working to develop single-cell metabolic technologies, the approach remains relatively new and faces numerous challenges, including the technical hurdle of how to collect cells without altering their metabolite abundance (126–129). To circumvent these issues, Miller et al. (130) developed a system that measures the activity of five key metabolic enzymes in conjunction with cell marker analysis to measure metabolic activity with single-cell resolution. Although this alternative approach is promising, there is no measure of global metabolic activity and the process is not high throughput. Thus, measuring metabolic activity at the single cell level, although highly promising for the study of GVHD, is currently limited by available technology.

Finally, the studies highlighted in this review have primarily utilized models using murine T cells and known strain combinations. While animal models have been integral to building foundational knowledge, their inherent limitations, including differences in murine and human immunology, pathogen free housing conditions, and genetic homogeneity can limit the translatability of the findings. Humanized murine models of xenogeneic GVHD (xGVHD) improve upon existing animal models by injecting human peripheral blood mononuclear cells (hPBMCs) into immunodeficient mice (131), allowing for the expansion and activation of human T cells *in vivo*. Increased use of xGVHD models will undoubtedly increase the likelihood that experimental findings will apply to human patients. However, xGVHD models themselves are imperfect and do not improve entirely upon deficiencies of classical animal models. Thus, patient samples may prove to be the best way to validate laboratory findings and ensure that observations are applicable to real world scenarios. In recent years, the repertoire of available technologies for *in vivo* human work has expanded, making the *in vivo* study of T cell metabolism during GVHD more feasible than ever. For example, recent publications have used administration of non-radioactive isotope tracers to study tissue metabolism in human patients in real time (132, 133). It could be envisioned that using non-radioactive isotope tracing in GVHD patients at the time of diagnosis could help to determine the dynamics of T cell metabolism during an active alloreactive immunologic response *in vivo*.

## Conclusion

**Figure 2:**
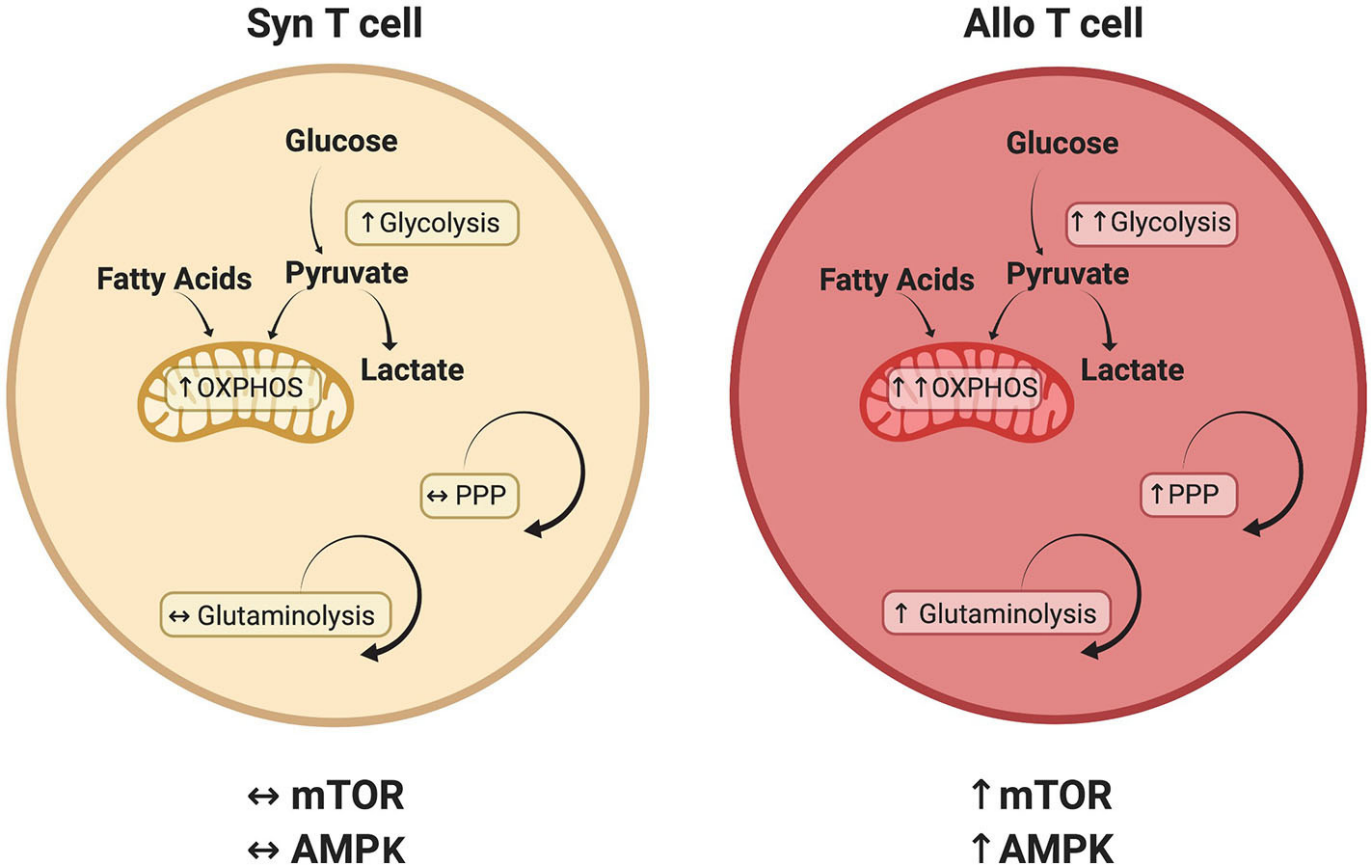
Metabolic pathways that distinguish alloreactive and syngeneic T cells. T cells isolated from allogeneic recipients have a unique metabolic profile including a greater increase in both glycolysis and oxidative phosphorylation (OXPHOS) (29, 53, 124). While various carbon sources contribute to this increased oxidative metabolism, including glucose (29, 52) and fatty acids (54), the relative contribution of each substrate remains to be determined. Glutaminolysis and the PPP are also preferentially upregulated in alloreactive T cells, as is activation of the AMPK and mTOR pathways (29, 70, 74). Because T cell metabolism is a dynamic process, this figure represents known or suspected metabolic activity in donor T cells on day 7 post-transplant. The relative contribution of each pathway is likely to change over time as discussed in the text.

This review has investigated recent developments in our understanding of alloreactive T cell metabolism. While evidence suggests that many metabolic pathways are active in alloreactive T cells (Figure 2), including glycolysis, OXPHOS, FAO, and glutamine metabolism, there is no current consensus on the relative importance of each pathway or their temporal necessity. Furthermore, while studies have examined the significance of glycolysis and OXPHOS independent from each other, it is likely that glycolysis and OXPHOS increase simultaneously in alloreactive cells to meet enhanced energetic demands. In addition, the inherent ability of T cells to exhibit metabolic flexibility, adopting a compensatory metabolism when an initial pathway is lesioned (as occurs with genetic deletions), coupled with the indirect and somewhat pleiotropic nature of metabolic activators and inhibitors, makes assignment of causality difficult in many cases. Finally, T_reg_ exhibit a distinct metabolic profile linking oxidative metabolism to T cell suppressive function, a phenomenon that appears to hold during allogeneic transplantation, yet remains the focus of intense investigation. In the end, studying T cell metabolism in the context of GVHD will help to deepen our understanding of *in vivo* T cell biology and identify novel therapies for the treatment of T cell-mediated pathologies.

## Author Contributions

RB reviewed the literature, drafted the manuscript, and reviewed the final product. CB reviewed and interpreted the primary literature, edited the manuscript draft, and critically revised the final manuscript. All authors contributed to the article and approved the submitted version.

## Conflict of Interest

The authors declare that the research was conducted in the absence of any commercial or financial relationships that could be construed as a potential conflict of interest.
